# Relaxin does not prevent development of hypoxia-induced pulmonary edema in rats

**DOI:** 10.1007/s00424-022-02720-9

**Published:** 2022-07-02

**Authors:** Ute Kowalleck, Mohamed A. Abdalla Ahmed, Julia Koedel, Katrin Schierle, Aida Salameh, Beate Rassler

**Affiliations:** 1 Carl-Ludwig-Institute of Physiology, University of Leipzig, Leipzig, Germany; 2 Institute of Pathology, University of Leipzig, Leipzig, Germany; 3 Department of Pediatric Cardiology, Heart Centre, University of Leipzig, Leipzig, Germany

**Keywords:** Relaxin, Normobaric hypoxia, Pulmonary edema, Pulmonary inflammation, Cardiovascular function

## Abstract

Acute hypoxia impairs left ventricular (LV) inotropic function and induces development of pulmonary edema (PE). Enhanced and uneven hypoxic pulmonary vasoconstriction is an important pathogenic factor of hypoxic PE. We hypothesized that the potent vasodilator relaxin might reduce hypoxic pulmonary vasoconstriction and prevent PE formation. Furthermore, as relaxin has shown beneficial effects in acute heart failure, we expected that relaxin might also improve LV inotropic function in hypoxia. Forty-two rats were exposed over 24 h to normoxia or hypoxia (10% N_2_ in O_2_). They were infused with either 0.9% NaCl solution (normoxic/hypoxic controls) or relaxin at two doses (15 and 75 μg kg^−1^ day^−1^). After 24 h, hemodynamic measurements and bronchoalveolar lavage were performed. Lung tissue was obtained for histological and immunohistochemical analyses. Hypoxic control rats presented significant depression of LV systolic pressure by 19% and of left and right ventricular contractility by about 40%. Relaxin did not prevent the hypoxic decrease in LV inotropic function, but re-increased right ventricular contractility. Moreover, hypoxia induced moderate interstitial PE and inflammation in the lung. Contrasting to our hypothesis, relaxin did not prevent hypoxia-induced pulmonary edema and inflammation. In hypoxic control rats, PE was similarly distributed in the apical and basal lung lobes. In relaxin-treated rats, PE index was 35–40% higher in the apical than in the basal lobe, which is probably due to gravity effects. We suggest that relaxin induced exaggerated vasodilation, and hence pulmonary overperfusion. In conclusion, the results show that relaxin does not prevent but rather may aggravate PE formation.

## Introduction

Exposure to acute hypoxia can induce pulmonary edema (PE) as can be observed after rapid ascent to high-altitude (so-called high-altitude PE, HAPE). Hypoxia-induced PE such as HAPE is characterized as a non-cardiogenic edema caused by elevated pulmonary capillary pressure [11, 43]. The increase in pulmonary capillary pressure is caused by hypoxic pulmonary vasoconstriction (HPV), which occurs both in pulmonary resistance arterioles and venules as one of the earliest events in the response to hypoxia [63]. In fact, subjects who are prone to develop HAPE showed enhanced HPV and an excessive rise in pulmonary artery systolic pressure (PASP) [32, 35]. Unevenness in the regional strength of HPV is considered an important factor in the formation of HAPE [34]. In areas with low HPV, regional overperfusion in combination with elevated venous resistance promotes water accumulation in the lung and finally, fluid filtration into pulmonary interstitium [71]. Regional heterogeneity of pulmonary perfusion in hypoxia may account for the patchy distribution of edema as demonstrated in chest radiographs or computerized tomography scans in HAPE patients [11]. Although hypoxia-induced PE is mainly a hydrostatic edema, it is accompanied by upregulation of inflammatory cytokines such as tumor necrosis factor alpha (TNFα) and inflammatory reactions in the lung [41, 44].

Many studies on HAPE-susceptible persons suggested that hypoxia impairs endothelial function, thus predisposing to greater vasoconstriction. Duplain and co-workers [28] reported a significant decrease in exhaled pulmonary nitric oxide (NO) at high altitude in HAPE-susceptible subjects compared to controls. A similar result was obtained in HAPE-susceptible subjects exposed to normobaric hypoxia for 2–4 h [15]. In subjects with HAPE, PASP decreased significantly following inhalation of NO [3, 59]. These results indicate that decreased bioavailability of NO plays an important role in the enhanced HPV [13]. Correspondingly, phosphodiesterase (PDE)-5 inhibitors such as sildenafil reduced PASP at high altitude by inhibition of degradation of cGMP, the second messenger of NO [31, 55]. However, inhalation of NO did not completely normalize PASP in HAPE-susceptible subjects [3], indicating that additional factors such as increased sympathetic activity [39, 47] or endothelin (ET) [58] contribute to the exaggerated HPV in these subjects. In healthy subjects exposed to normobaric hypoxia for 90 min, a single dose of the ET receptor antagonist bosentan significantly blunted the hypoxia-induced increase in PASP [51]. Correspondingly, pulmonary vasodilators, in particular the L-type calcium channel antagonist nifedipine or PDE-5 inhibitors such as sildenafil or tadalafil, are recommended in the treatment of HAPE [37, 43].

In recent years, the insulin-related peptide relaxin (RLX) has gained new attention as a potent vasodilating and cardioprotective agent. RLX has originally been regarded as a pregnancy hormone as it is mainly produced by the corpus luteum, the uterus, and the placenta and reaches its highest levels during pregnancy [7]. However, RLX is also expressed in other tissues such as the heart and vessels. In humans, RLX has been observed to be markedly upregulated in congestive heart failure [26]. In various disease models in rats, RLX reduced cardiac contractile dysfunction and protected the heart from inflammation, hypertrophy, and fibrosis [6, 72]. RLX has been shown to promote NO synthesis and to inhibit ET-1 release in endothelial cells [8, 25]. RLX also proved to protect lungs from ischemia–reperfusion injury via reduction of ET-1 and induction of inducible NO synthase (iNOS), thus counteracting vasoconstriction and attenuating pulmonary edema [2].

In previous studies on rats exposed to normobaric hypoxia, we had observed development of pulmonary injury characterized by edema and inflammation, which emerged after 6 h and peaked after 16–24 h of hypoxia [14, 54]. It was accompanied by a depression of inotropic LV function [4, 14]. The present study was conducted to examine whether the vasodilator RLX might attenuate or even prevent formation of hypoxia-induced pulmonary injury. In rats, PE develops within the first 24 h of exposure to normobaric hypoxia and is accompanied by inflammation. Similarly, depression of LV inotropy occurred after 6 h of hypoxia and showed a tendency to recovery after 24 h of hypoxia [14]. Therefore, we chose this interval of time to study the effects of concomitant RLX infusion and hypoxia on development of pulmonary injury and on cardiac function in rats. Due to its positive effects on pulmonary vasodilation and cardiac contraction, we hypothesized that RLX would counteract both hypoxia-induced pulmonary injury and LV depression.

## Materials and methods

### Animal model

All experiments were performed on 42 female Sprague–Dawley rats supplied by Charles River (Sulzfeld, Germany). The body weight was 225 ± 2.2 g at the beginning of the study corresponding to an age of about 12 weeks. All animal protocols were approved by the Federal State Agency. The experiments were conducted in accordance with the Guide for the Care and Use of Laboratory Animals published by the National Institutes of Health and with the “European Convention for the Protection of Vertebrate Animals used for Experimental and other Scientific Purposes” (Council of Europe No 123, Strasbourg 1985).

### Study protocol

Animals were exposed to normoxia (*N*) or normobaric hypoxia (*H*) for 24 h. For exposure to hypoxia, the animals were placed into a hypoxic chamber sized 65 × 105 × 50 cm. The gas mixture in the chamber contained 10% oxygen in nitrogen. A special equipment prevented penetration of ambient air during manipulations on the animals, thus keeping the oxygen concentration in the chamber stable at 10 ± 0.5%. Normoxic animals remained under room air condition. Additionally, all animals received an intravenous infusion over the total experimental time. Normoxic (NCtrl, *n* = 8) and hypoxic controls (HCtrl, *n* = 8) were infused with 0.9% sodium chloride (NaCl) solution. Two doses of human recombinant relaxin-2 (RLX) were studied in hypoxic animals, a low dose (HRLX-L: 15 μg kg^−1^ day^−1^, *n* = 10) and a high dose (HRLX-H: 75 μg kg^−1^ day^−1^, *n* = 8). For control, a normoxic group received the low RLX dose (NRLX-L, *n* = 8). RLX was obtained from Novartis (Basel, Switzerland).

Infusions were administered with automatic pumps (Infors AG, Basel, Switzerland) at a rate of 0.1 ml h^−1^ via an infusion catheter (Vygon, Aachen, Germany). The infusion catheter was inserted into the left jugular vein. This operation was performed in 2% isofluran anesthesia. After catheter insertion, the animals woke up and moved freely with access to tap water and rat chow diet (Altromin C100, Altromin GmbH, Lage, Germany). Exposure to hypoxic environment started immediately after catheter insertion.

### Hemodynamic measurements

About 30–40 min before the end of the exposure time, the animals were anesthetized with thiopental (Trapanal® 80 mg kg^−1^, i.p.). They were tracheotomized, and a polyethylene cannula was placed in the trachea. The right ventricle (RV) and left ventricle (LV) were catheterized with Millar® (Millar Instruments, Houston, TX) ultraminiature catheter pressure transducers (for more details see our previous publication [52]) to measure LV and RV systolic pressures (LVSP, RVSP) and heart rate (HR). In addition, LV and RV maximal velocities of increase (dP/dtmax) and decrease in pressure (dP/dtmin) were determined as measures of ventricular contractility and relaxation, respectively. After withdrawal of the LV catheter tip into the aorta, diastolic aortic pressure (DAP) was measured to calculate mean aortic pressure (MAP). Cardiac index (CI, body mass-related cardiac output) was determined by thermodilution using a thermosensitive 1.5F microprobe and a Cardiomax II computer (Columbus Instruments, Columbus, OH). The total peripheral resistance (TPR) was calculated by dividing MAP by CI. Hypoxic animals remained in hypoxia until completion of hemodynamic measurements.

### Sampling of materials

After the hemodynamic measurements, the abdominal cavity was opened by midline incision. Animals were sacrificed by drawing blood from the abdominal aorta. After opening of the thoracic wall and ligation of the right main bronchus, a bronchoalveolar lavage (BAL) was performed two times consecutively with 3 ml 0.9% NaCl each. The fluid was instilled via the tracheal cannula into the left lung and withdrawn immediately. The recovery rate was about 90% on average. The left lung was discarded thereafter. From the intact right lung, tissue samples of the apical and basal lobes were fixated in formalin for histological analysis. A piece of the middle lobe was taken for determination of wet-to-dry weight (*W*/*D*) ratio. The blood was centrifuged for 10 min at 2100 r.p.m. Serum and recovered BAL fluid were frozen and stored at − 80 °C for further analyses.

### RLX concentration in serum

Circulating RLX was analyzed in EDTA-containing serum of RLX-infused rats using Human Relaxin-2 Quantikine® ELISA Kit (R & D Systems, Minneapolis, MN) according to the manufacturer’s instructions. The assay is specific for human relaxin-2, which means, it does not significantly cross-react with rat RLX. The sensitivity of the assay is ~ 7.81 pg/ml (equivalent to the lowest standard), the maximum dose for accurate determination was 500 pg/ml as reported by R & D Systems. Therefore, serum samples were diluted 1:10–1:50 with phosphate-buffered saline (PBS), so that values would fall on the standard curve. Optical density was measured at 570 nm using a SpectraCount™ (Packard Instrument Company Inc., Meriden, CT). Serum concentrations [ng/ml] were obtained after correction of the readings by the zero standard and multiplication with the dilution factor.

### Lung histology

The formalin-fixated tissue samples of the right lung were embedded in paraffin, sliced, and stained with hematoxylin–eosin. Histological assessments were done by two independent investigators (UK and JK or KS), who were blinded towards the treatment group. In the lung, they evaluated PE, inflammation, and congestion. For a detailed quantification of PE, the complete histological section of a lung was assessed. First, PE severity in each area of the section was gauged visually by evaluating the width of alveolar septa and the definition of alveolar spaces. PE scores ranged from 0 (absent), 1 (mild: alveolar septa slightly thickened, alveolar space well defined), 2 (moderate: thickness of alveolar septa about double the normal width, alveolar space narrowed but still defined), to 3 (severe: alveolar spaces hardly determinable and/or alveolar edema). The PE index (PEI) was calculated by cumulating the products of PE score and proportionate area of each part of the histological preparation. The congestion index (ConI) was determined in analogous way. For a more detailed examination of PE distribution, the PE indices of the apical (AL) and basal lung lobes (BL) were differentially analyzed.

### Immunohistochemistry

The expression of TNFα in the lung was determined using immunohistochemistry. Specimens from the apical lobes of the right lung, fixed with 4% formalin, were embedded in paraffin, and 1-μm slices were cut. After mounting on microscopic slides, the samples were dewaxed and rehydrated. Antigen retrieval was performed according to Dhein et al. [24]. Thereafter, lung tissue was incubated with the primary antibody for TNFα, (goat polyclonal IgG, dilution 1:100, Santa Cruz, Heidelberg, Germany) at 4 °C over night. Afterwards, the slides were washed and the appropriate anti-goat secondary antibody labeled with horseradish peroxidase was applied for 1 h at room temperature. Subsequently, the specimens were washed again, and visualization of positive cells was performed with 3-amino-9-ethylcarbazole (AEC, red chromogen, Dako, Hamburg Germany). Cell nuclei were counterstained with hematoxylin. The specimens were investigated microscopically using the Axioimager M1 microscope from Zeiss (Carl Zeiss, Jena, Germany). TNFα is mainly located in the bronchial and peribronchial regions. Therefore, photographs were taken from these regions using the AxioCam MRc 5 camera and the AxioVision Rel. 4.6 software (Carl Zeiss, Jena, Germany) at 10 × magnification. At least 10 pictures per animal were evaluated by a blinded observer (UK). For measurements in the pictures, the program ImageJ [1] was used.

As TNFα is located within the cytosol, we determined the TNFα-positive area (given in µm^2^). For quantifying the expression of TNFα, we analyzed the intensity (integrated density given in arbitrary units [a.u.]) divided by the TNFα-positive area.

### Lung wet-to-dry weight ratio

Lung tissue samples were weighed immediately after preparation (wet weight, *W*) and after drying in an oven at 75 °C for 48 h (dry weight, *D*). The *W*/*D* ratio served as a surrogate parameter of water accumulation in the lung.

### Protein concentration in serum and BAL fluid

Total protein concentration in serum and BAL fluid was determined using the Bradford method. For BAL fluid analysis, the undiluted supernatant of the first lavage was used. Serum was diluted with PBS at a ratio of 1:250. We applied an Advanced Protein Assay (Cytoskeleton Inc., Denver, Colorado, USA) using Coomassie brilliant blue G-250 as the dye and bovine serum albumin (BSA) as the protein standard. The absorbance was immediately measured at 570 nm using the SpectraCount™ (Packard Instrument Company Inc., Meriden, Connecticut, USA). Protein concentrations of the samples were calculated by comparing the sample absorbances with those of a standard curve using I-smart (Packard Instrument Company Inc., Meriden, Connecticut, USA). For generation of the standard curve, a four-parametric test was used. The protein concentrations that could be determined reliably ranged from 25 to 600 µg ml^−1^. Protein concentrations in serum ([P]_S_) and in BAL fluid ([P]_L_) are given in μg/ml. The concentration ratio [P]_L_/[P]_S_ was calculated for each individual animal and is given in %.

### Statistical analysis

Statistical analyses were carried out with the software package SigmaPlot Version 14.0 (Systat Software GmbH, Erkrath, Germany) for Windows. All groups were statistically compared using analysis of variance (ANOVA) procedures. At first, we performed a Shapiro–Wilk test of normality. If data were normally distributed, we used a one-way ANOVA with a post hoc test according to Fisher’s LSD method. These data are presented as means ± SEM. If the data were not normally distributed, a Kruskal–Wallis ANOVA on ranks with a post-hoc test according to Dunn’s method was applied. These data are given as medians [25^th^/75^th^ percentile]. Both post hoc tests are multiple comparison procedures comparing all possible pairwise mean differences. *P* values < 0.05 were considered significant.

## Results

### Serum RLX concentration

In hypoxia, low-dose RLX infusion increased to about twice the level of normoxic animals receiving the same RLX dose (1.2 ± 0.3 vs. 0.6 ± 0.1 ng/ml, *p* = 0.09). Infusion with the high RLX dose in hypoxia significantly increased serum RLX concentration to 12.3 ± 3.9 ng/ml (*p* = 0.004 compared to NRLX-L and *p* = 0.002 compared to HRLX-L).

### Hemodynamic results

**Fig. 1:**
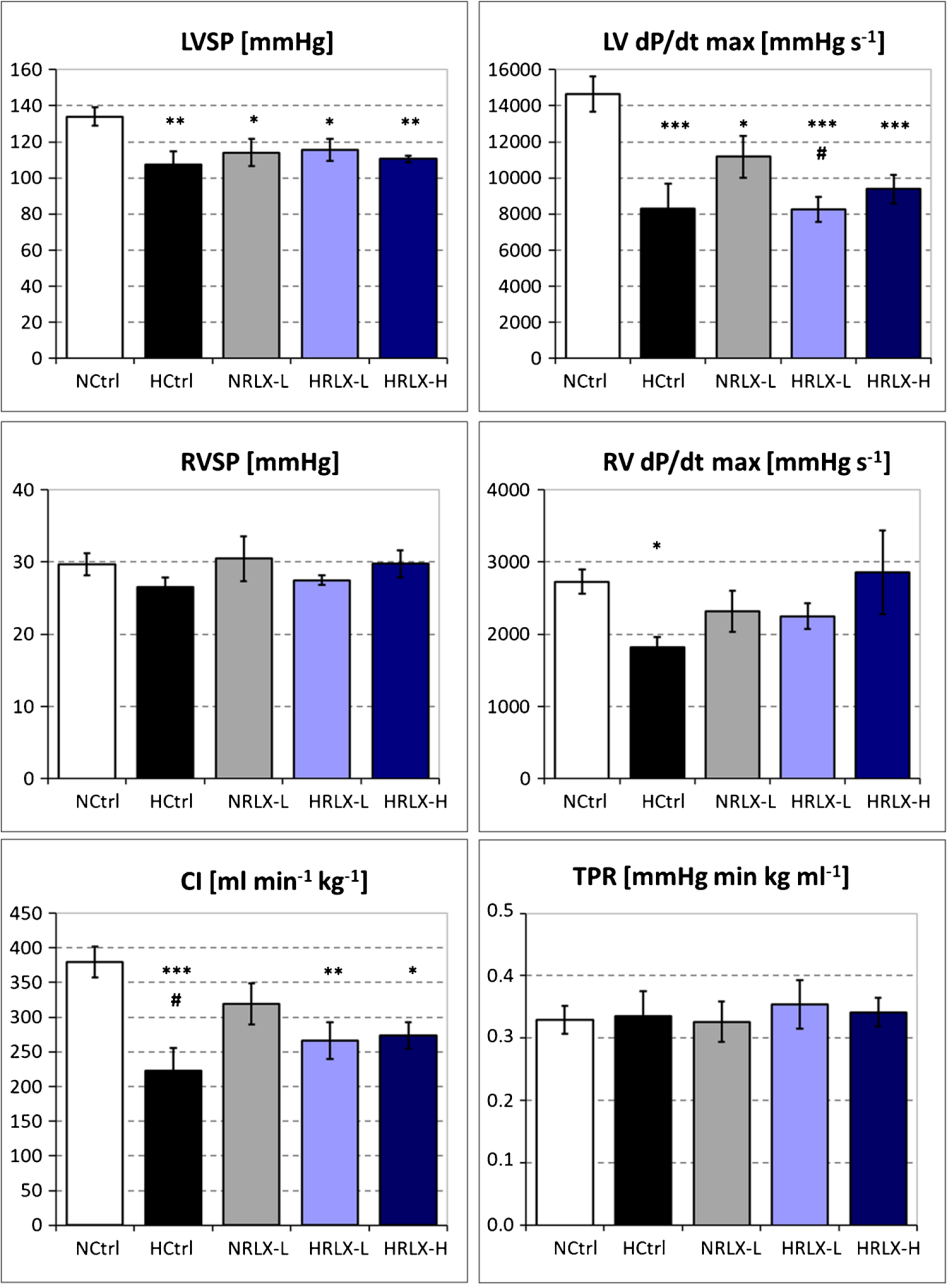
Hemodynamic results. LVSP, left ventricular systolic pressure (*n* = 7–8); LV dP/dtmax, left ventricular contractility (*n* = 7–8); RVSP, right ventricular systolic pressure (*n* = 7–9); RV dP/dtmax,right ventricular contractility (*n* = 7–9); CI, cardiac index (*n* = 6–8); TPR, total peripheral resistance (*n* = 6–7). Data is given as mean ± SEM. Significant differences vs. NCtrl: * *p* < 0.05; ** *p* < 0.01; *** *p* < 0.001; significant differences vs NRLX-L: # *p* < 0.05

Main hemodynamic results are shown in Fig. 1 and Table 1.

**Table 1:** Hemodynamic parameters

Group Parameter	NCtrl (*n* = 7–8)	HCtrl (*n* = 7–8)	NRLX-L (*n* = 7–8)	HRLX-L (*n* = 7–10)	HRLX-H (*n* = 7–8)
LVdP/dtmin [mmHg s^−1^]	− 11,228 ± 1185	− 9571 ± 1409	− 11,891 ± 1274	− 9842 ± 1550	− 9787 ± 947
RVdP/dtmin [mmHg s^−1^]	− 2012 ± 174	− 1620 ± 148	− 1913 ± 243	− 1682 ± 133	− 2064 ± 234
DAP [mmHg]	107.4 [100.6/112.5]	80.8 [61.4/90.8]	91.5 [80.2/100.7]	92.5 [62.2/104.4]	76.7* [72.2/82.9]
MAP [mmHg]	120.2 [115.3/125.4]	94.6 [74.8/104.3]	106.2 [90.3/114.1]	105.5 [75.7/117.8]	91.9* [88.0/96.1]
HR [min^−1^]	457 [416/497]	406 [358/431]	475 [347/487]	439 [399/453]	457 [446/482]

*NCtrl,* *HCtrl,* normoxic and hypoxic controls, respectively (saline infusion); *NRLX-L,* *HRLX-L, *normoxic and hypoxic animals, respectively, with RLX infusion (low dose = 15 μg kg-1 d-1); *HRLX-H*, hypoxic animals with RLX infusion (high dose = 75 μg kg-1 d-1). LV and RVdP/dtmin, left and right ventricular relaxation velocity; DAP, diastolic aortic pressure; MAP, mean arterial pressure; HR, heart rate. LV and RVdP/dtmin are given as mean ± SEM; DAP, MAP and HR are given as medians [25^th^/75^th^ percentile]. * marks a significant difference to NCtrl (*p*< 0.05)

#### LV function and systemic circulation

Hypoxia induced a significant depression of LV inotropy. After 24 h of hypoxia, LVSP decreased from 134 ± 5 to 108 ± 7 mmHg (*p* = 0.004). LV contractility (LV dP/dtmax) decreased significantly by more than 40% (NCtrl 14,657 ± 974 mmHg s^−1^ vs. HCtrl 8291 ± 1397 mmHg s^−1^; *p* < 0.001). Consequently, cardiac index (CI) was significantly reduced in hypoxia (NCtrl 380 ± 22 ml min^−1^ kg^−1^ vs. HCtrl 223 ± 33 ml min^−1^ kg^−1^; *p* < 0.001; Fig. 1). LV relaxation (LV dP/dtmin) decreased only slightly by about 15% (n.s.). TPR and DAP as indicators of vascular dilatation did not significantly change under hypoxia even though DAP and consequently, MAP decreased by more than 20% under hypoxia (Table 1).

RLX infusion in hypoxia did not improve LV inotropic depression neither at the low nor the high dose (Fig. 1). With low-dose RLX infusion both in normoxia and hypoxia, DAP and MAP remained at control level, but decreased significantly in hypoxia with high-dose RLX infusion. Of note, low-dose RLX infusion in normoxia induced a significant decrease of LVSP and LV dP/dtmax compared to NCtrl (by 15% and 24%, respectively). LVSP was on a similar level as in all hypoxic groups, but LV dP/dtmax was significantly higher than in the HRLX-L group. CI, DAP, and MAP were also mildly reduced in the NRLX-L group, but this was not significant (Fig. 1 and Table 1).

#### RV function and heart rate

RVSP remained largely stable in all groups, but RV contractility (RV dP/dtmax) decreased significantly under hypoxic conditions (NCtrl 2876 ± 178 mmHg s^−1^ vs. HCtrl 1815 ± 142 mmHg s^−1^; *p* = 0.016; Fig. 1), while RV relaxation (RV dP/dtmin) was only slightly reduced (n.s., Table 1). In normoxic animals, low-dose RLX infusion also decreased RV dP/dtmax, but this effect was only half as much as that of hypoxia. Hypoxia plus low-dose RLX infusion improved RV dP/dtmax, and in hypoxia at the high RLX dose, RV dP/dtmax was completely restored (Fig. 1). HR showed similar responses to hypoxia and/or RLX infusion. HR decreased slightly but not significantly in hypoxia. While RLX in normoxic animals had no remarkable effect on HR, it restored (HRLX-L) or even slightly improved (HRLX-H) HR in hypoxic animals (Table 1).

### Pulmonary injury

**Fig. 2:**
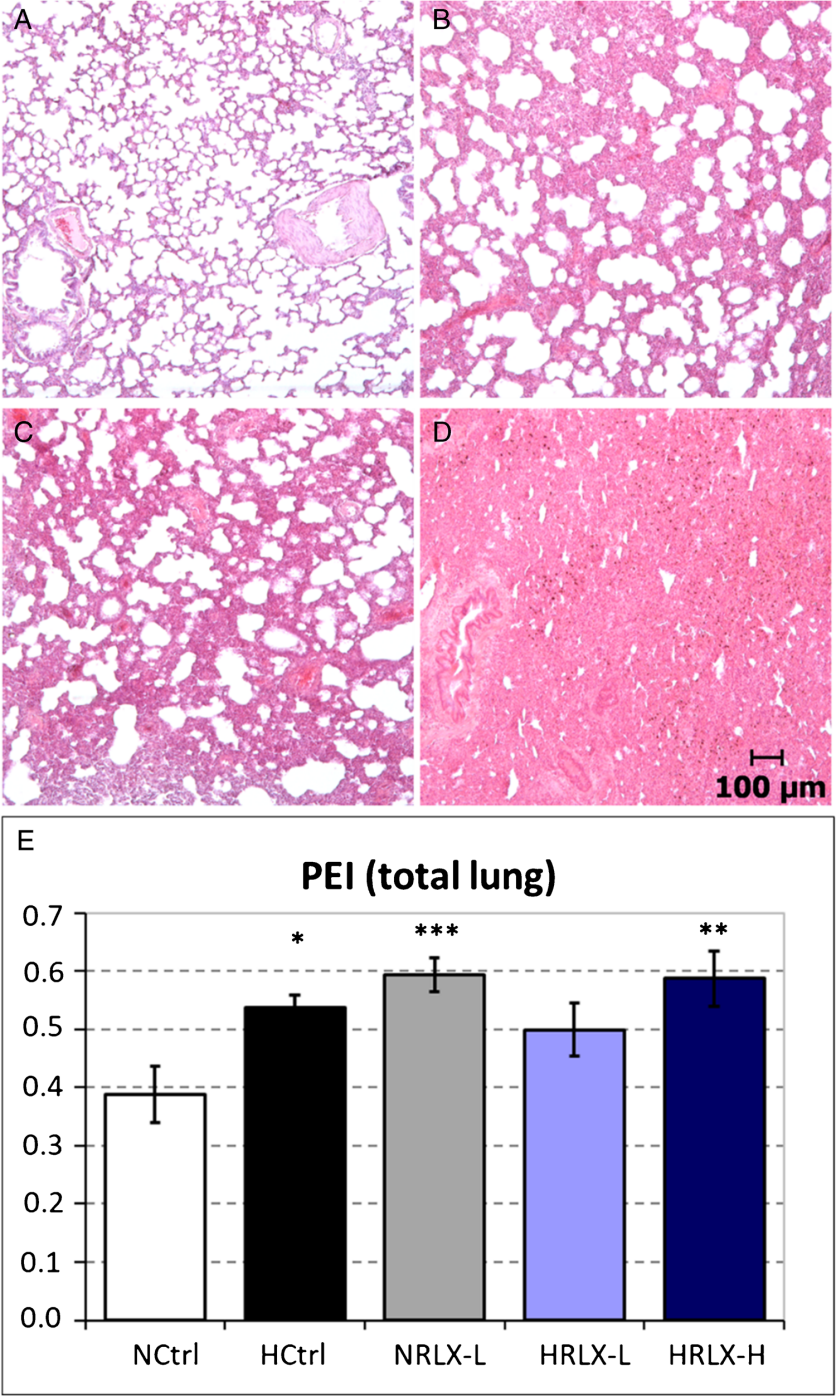
Lung histology. **A** NCtrl, normoxic control: normal lung tissue without edema. **B** HCtrl, hypoxic control: moderate interstitial edema. **C** HRLX-L, hypoxia + 15 μg RLX kg^−1^ day^−1^: moderate interstitial edema. **D** HRLX-H, hypoxia + 75 μg RLX kg^−1^ day^−1^: severe interstitial edema. All slices (A, B C, D) are stained with hematoxylin–eosin; original magnification 5 × . Histologic examples for the normoxic RLX-L group are given in Fig. 3. **E** Pulmonary edema index (total lung). Data is given as mean ± SEM, *n* = 8–10. Significant differences vs. NCtrl: * *p* = 0.017; ** *p* = 0.002; *** *p* = 0.001

In the normoxic controls, there were no or only mild histologic signs of PE (PEI = 0.39 ± 0.05, Fig. 2A). After 24 h of normobaric hypoxia, PEI was significantly higher (0.54 ± 0.02, *p* = 0.017 compared to normoxic controls), indicating a moderate interstitial PE (Fig. 2B). With low-dose RLX infusion in hypoxia, PEI decreased slightly to a level that was not significant compared to normoxic controls (0.50 ± 0.05, *p* = 0.06; Fig. 2C). However, infusion with the high RLX dose aggravated PEI (0.59 ± 0.05; *p* < 0.002 compared to normoxic controls; Fig. 2D). Of note, RLX infusion in normoxia induced a similar degree of PE, and this was more severe than PE in hypoxic controls (0.59 ± 0.03; *p* < 0.001 compared to NCtrl; Fig. 2E).

**Fig. 3:**
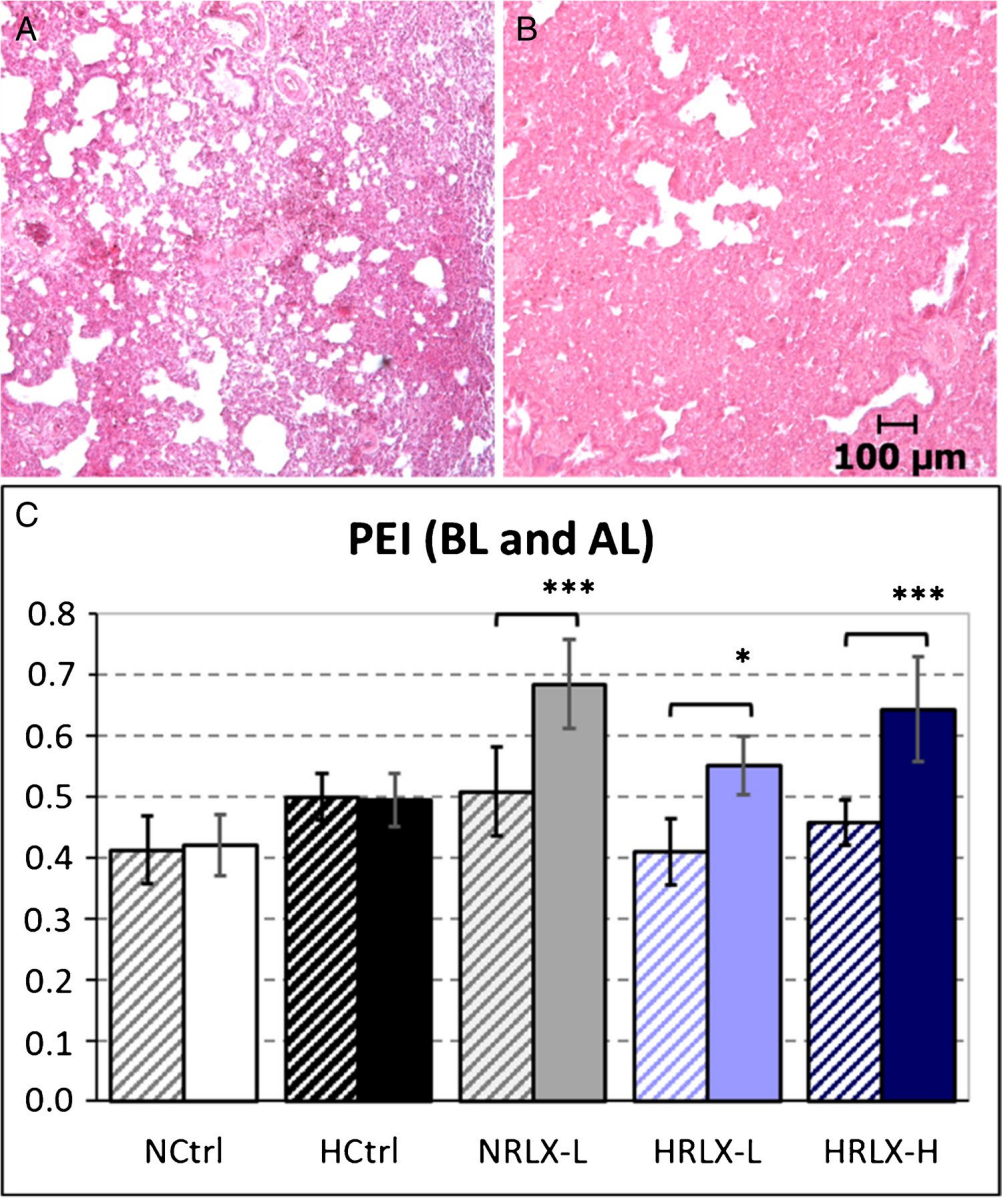
Lung histology, comparison basal lobe (BL) vs. apical lobe (AL). **A** NRLX-L, normoxia + 15 μg RLX kg^−1^ day^−^.^1^), basal lobe: moderate interstitial edema. **B** NRLX-L (same animal as in (A)), apical lobe: severe interstitial edema. The slices (A, B) are stained with hematoxylin–eosin; original magnification 5 × . **C** Pulmonary edema index determined in basal lobe (BL  ) and apical lobe (AL  ). Data is given as mean ± SEM, *n* = 8–10. Significant differences vs. NCtrl: * *p* = 0.025; *** *p* < 0.001; significant differences AL vs. BL: *p* < 0.05

A more detailed analysis of PE distribution over the lungs revealed a very interesting finding: While in both normoxic and hypoxic controls, PEI was similar in the apical (AL) and basal lung lobes (BL), we observed marked and significant differences in animals after RLX infusion. PE was particularly prominent in the AL of RLX-infused animals. In the AL of both normoxic and hypoxic RLX animals, PEI was 35–40% higher than in the BL (*p* < 0.05; Fig. 3).

**Fig. 4:**
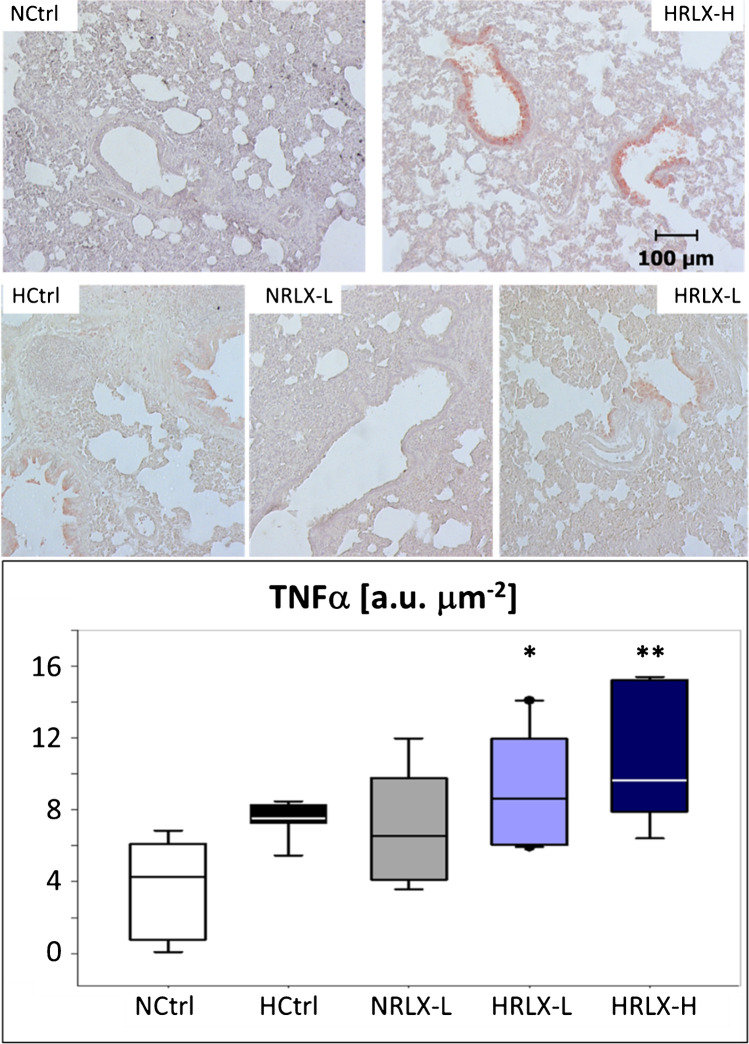
TNFα expression in lung tissue. Upper panels, left: NCtrl, normoxic control: normal lung tissue without inflammation; right: HRLX-H, hypoxia + 75 μg RLX kg^−1^ day^−1^: marked peribronchial expression of TNFα. Middle panels, left: HCtrl, hypoxic control: mild-to-moderate expression of TNFα; mid: NRLX-L, normoxia + 15 μg RLX kg^−1^ day^−1^: mild expression of TNFα; right: HRLX-L, hypoxia + 15 μg RLX kg^−1^ day^−1^: mild-to-moderate expression of TNFα. All slices: original magnification 10 × . Lower panel: TNFα expression (in a.u./µm^2^). Data is given as medians (lines in the boxes) with 25^th^/75^th^ percentiles (boxes), 10^th^/90.^th^ percentiles (whiskers), and outliers (circles); *n* = 7–10. Significant differences vs. NCtrl: * *p* = 0.014; ** *p* = 0.002

PE was accompanied by inflammatory reactions. Immunohistochemical analysis of the lungs showed a significant increase in TNFα expression in hypoxic rats, which was mainly located in the bronchial and peribronchial regions. Treatment with RLX did not mitigate but rather promoted this inflammatory reaction as indicated by comparison of NRLX-L and HRLX-H rats with their respective controls (Fig. 4).

Lung histology revealed presence of congestion in the lungs as assessed by congestion index (ConI). Hypoxia alone did not cause congestion (ConI: NCtrl 0.25 [0/1]; HCtrl 0.25 [0/0.87]), but RLX infusion, both in normoxia and hypoxia, induced congestion. In the lungs of RLX animals, congestion was not evenly distributed but showed marked regional differences. ConI was most pronounced in NRLX-L (1.0 [0.81/1.81]) compared to hypoxic RLX groups (HRLX-L 0.75 [0/1.25]; HRLX-H 0.87 [0.06/1.0]), but the differences among groups were not significant. Accordingly, lung *W*/*D* ratios of RLX-infused animals were elevated (4.99 [4.79/5.52], 5.09 [4.89/5.17], 4.96 [4.84/5.20] in NRLX-L, HRLX-L, and HRLX-H, respectively) compared to normoxic (4.89 [4.72/4.97]) and hypoxic controls (4.93 [4.66/5.05]), but the differences among the groups were also not significant.

To investigate whether capillary wall stress might have aggravated PE and whether RLX might attenuate it, we determined protein concentration in BALF ([P]_L_) and in serum ([P]_S_) as well as the ratio between them ([P]_L_ / [P]_S_). The concentration ratio [P]_L_ / [P]_S_) increased slightly both in HCtrl rats and in animals with low-dose RLX infusion (NRLX-L, HRLX-L), but the differences were not significant. The increase was mainly due to a significantly elevated protein content in BALF ([P]_L_) in these groups, indicating that hypoxia and/or low-dose RLX infusion may promote disruption of capillary walls and alveolar protein leakage. Of note, [P]_S_ significantly increased in HRLX animals, indicating a reduction of plasma volume. Notably, with RLX infusion at the high dose, [P]_L_ was almost at control level suggesting that reduction of plasma volume might have prevented disruption of capillary walls, and hence, transition into alveolar edema (Table 2).

**Table 2:** Protein concentration in BAL fluid and serum

Group	NCtrl (*n* = 6)	HCtrl (*n* = 7)	NRLX-L (*n* = 8)	HRLX-L (*n* = 9)	HRLX-H (*n* = 7)
[P]_L_ [μg ml^−1^]	473 [366/694]	777 * [659/1132]	815 * [599/947]	924 * [716/1107]	610 [526/895]
[P]_S_ [μg ml^−1^]	88,042 [65762/93496]	99,397 [60990/143783]	98,303 [81709/116812]	115,743 * [98810/140564]	131,691 * [94268/141703]
[P]_L_/[P]_S_ [%]	0.69 [0.34/0.99]	0.86 [0.45/1.43]	0.85 [0.74/1.09]	0.81 [0.55/1.05]	0.52 [0.47/0.80]

*NCtrl, HCtrl*, normoxic and hypoxic controls, respectively (saline infusion); *NRLX-L, HRLX-L*, normoxic and hypoxic animals, respectively, with RLX infusion (low dose = 15 μg kg^−1^ day^−1^); *HRLX-H*, hypoxic animals with RLX infusion (high dose = 75 μg kg^−1^ day^−1^). *[P]*_*L*_, protein concentration in BAL fluid [μg ml^−1^]; *[P]*_*S*_, protein concentration in serum [μg ml^−1^]; *[P]*_*L*_*/[P]*_S_, ratio of protein concentration BAL fluid/serum (given in %). Data is given as median [25^th^/75.^th^ percentile]. Significance marks: * significant vs. NCtrl (*p* < 0.05)

## Discussion

The main finding of the present study was that infusion of the vasodilator RLX did not prevent formation of hypoxia-induced PE. With the low dose of 15 μg kg^−1^ day^−1^, slight attenuation was achieved while the high dose (75 μg kg^−1^ day^−1^) even aggravated PE. To the best of our knowledge, this is the first study investigating effects of RLX on formation of hypoxia-induced PE in rats. Recombinant human relaxin has been shown to reduce hypoxic pulmonary hypertension in rats [66]. These authors infused doses of 50 and 240 μg kg^−1^ day^−1^ over 10 days into rats exposed to normobaric hypoxia. With these doses, they attained serum levels of RLX similar to levels of early pregnancy rats [66], which are in the range of 20–40 ng ml^−1^. Danielson and co-workers investigated RLX effects on renal function by administering various RLX doses between 0.04 and 40 μg h^−1^ over 48 h producing serum RLX concentrations ranging from 1 to 80 ng ml^−1^. With their most effective RLX dose of 0.4 μg h^−1^, they attained a serum concentration of about 11 ng ml^−1^ [19]. This dose induced a similar effect as an RLX dose of 4 μg h^−1^ producing serum concentrations of 20 ng ml^−1^ after 48 h and 7 ng ml^−1^ after 6 h of infusion [19, 20]. Noteworthy, infusion at 40 μg h^−1^ over 48 h (serum concentration 80 ng ml^−1^) proved to be ineffective [19]. This bell-shaped RLX effect corresponds with our finding that the high RLX dose did not attenuate PE. The reasons for this bell-shaped dose–response curve are not completely clear. Several mechanisms are discussed, e.g., that ligand dissociation is accelerated at higher RLX concentrations, thus shortening the time of RLX binding to its receptor [62]. As another possible explanation, different dose–response curves for venous and arterial dilator properties of RLX are discussed [27].

The low RLX dose applied in our experiments induced serum concentrations of 0.6 and 1.2 ng/ml in normoxia and hypoxia, respectively, which were associated with vasodilatory effects as discussed in more detail below. It seems surprising that the same infusion dose of RLX induced such a large difference in RLX serum concentration between normoxic and hypoxic animals. Administered RLX is removed from the blood by uptake into several tissues and sequestration as well as by degradation and clearance [29]. These processes, particularly enzymatic degradation and clearance, may be compromised in hypoxia, which may account for the higher RLX serum concentrations in hypoxic rats. Moreover, previous studies have shown that serum concentrations of RLX are not in linear correlation with the infused RLX dose [19]. High doses of RLX may cause hypovolemia due to its stimulating effect on glomerular filtration [23], which may account for the disproportionate increase in serum RLX concentration in the HRLX-H group.

### Hemodynamic effects

Hypoxia over 24 h induced a significant drop in LV inotropic function (LVSP, LV contractility), a mild reduction in HR and DAP and consequently, a significant decrease in CI. This is in line with previous findings [14]. Animal studies showed that hypoxia induced a depression of LV contractility due to a decrease in myocardial oxygen consumption [68]. In addition, hypoxia compromises mitochondrial respiration and consequently, ATP synthesis in the rat LV [5, 33]. In our experiments, RLX did not improve the hypoxic depression of LV inotropy. This corresponds to the findings of other studies in rodents that revealed no evidence for a positive inotropic action of RLX infusion on the ventricles [42, 56] but only on the atria [38]. Moreover, our results showed that RLX depressed LVSP and LV contractility even in normoxia. Several factors may account for the absence of a positive or even a negative inotropic effect of RLX. The reduction of LVSP might result from the vasodilatory effect of RLX on many vascular beds. At basal doses, RLX produces mostly venous vasodilation, while arterial vasodilation is mainly achieved with higher RLX doses [27]. This may have reduced venous return and consequently, LV end-diastolic filling. To clarify this assumption, further research is necessary, in particular, measurement of volume changes and recording of pressure–volume loops. Another possible explanation is related to increased NO production, which is a pivotal mechanism of action for RLX [2, 8]. Of note, vasodilation is not the only NO effect on the cardiovascular system. NO is involved in the regulation of cardiac contraction, oxygen consumption, and mitochondrial function. High amounts of NO may have negative inotropic effects in the normal heart [46]. In hypoxic cardiomyocytes, high levels of NO result in the formation of peroxynitrite, a reactive nitrogen species with high cytotoxicity, which compromises mitochondrial function and oxygen consumption [21]. These detrimental effects seem to contradict many reports on cardioprotective effects of RLX and deserve more detailed research.

Nevertheless, many of the cardioprotective effects of RLX observed in animals and humans are associated with the vasodilatory properties of RLX [8, 18, 26]. Moreover, RLX has antiapoptotic, antihypertrophic, antiinflammatory, and antifibrotic effects [45, 48, 57]. In in vivo experiments on rats, these effects were attained after chronic RLX infusion over 2 weeks [42, 45, 69]. RLX infusion at 4 μg h^−1^ over 10 days in rats induced a significant increase in stroke volume and cardiac output. The increase of stroke volume became significant as early as 6 days after start of infusion while mean arterial pressure showed no significant increase [22]. With RLX infusion at the same RLX dose, RLX serum concentrations increased with longer duration of infusion [19, 20]. Therefore, we think that beneficial effects of RLX on the heart can occur after chronic administration as suggested by results of clinical studies in heart failure patients showing improved patient survival after 6 months of treatment [65].

A vasodilatory effect of RLX was found in our study as reflected by a decrease of DAP and MAP. Vasodilation may explain, at least in parts, the CI reduction with RLX infusion in normoxia and even more in hypoxia. One would expect TPR to decrease due to vasodilation. However, TPR is a secondary parameter calculated from the primary parameters CI and blood pressure amplitude. If CI is reduced due to the Frank-Starling mechanism, calculated TPR is consequently increased, and this may mask vasodilatory effects on TPR. In addition, HR was restored by RLX infusion in hypoxia. This is in correspondence with earlier findings in the rat heart [38]. However, this mild positive chronotropic effect of RLX was not able to fully compensate for the reduction in LV inotropy.

Compared to LV, hypoxic effects on systolic pressure and contractility of the RV were milder. In previous experiments, we observed no reduction of RVSP and RV dP/dtmax after short-term (1.5–16 h) exposure to hypoxia and even a mild but not significant improvement after 24 h [14]. In contrast to its effects on LV, RLX improved RV inotropic function in hypoxia at the low dose and even restored it at the high dose. Although there is a close mechanical and functional interdependence between the RV and the LV, the two cardiac ventricles show many differences in their responses to adverse loading such as administration of adrenergic agonists [30]. In a rat model with norepinephrine (NE) infusion, the inotropic response of the RV was significantly higher than that of the LV [36]. While NE infusion increased RVSP and RV dP/dtmax more than two-fold, LVSP remained at control level and LV dP/dtmax mildly increased by about 50%. Combined α- and β-adrenergic blockade with carvedilol completely abrogated the inotropic effects of NE [10]. In non-pregnant rats, RLX administration has been shown to increase sympathetic nerve activity [17]. Thus, enhanced sympathetic activation and its differential effects on the cardiac ventricles might be an explanation for the differences in the RV and LV inotropic responses to RLX. Furthermore, cardiomyocyte hypertrophy and cardiac stiffness are promoted by reduced NO levels and compromised NO signalling in cardiomyocytes and can be counteracted by PDE-5 inhibition [64]. PDE-5 has been found upregulated in hypertrophied RV myocardium of humans and rats, and acute inhibition of PDE-5, which results in increased cGMP levels, improved contractility of the RV but not of the LV [49]. Probably, RLX via its stimulatory effect on NO synthesis may have a similar effect on RV contractility as exerted by PDE-5 inhibitors.

### Pulmonary effects

Normobaric hypoxia over 24 h induced development of moderate interstitial PE, which was accompanied by inflammation as expressed by a significant increase in TNFα expression in the lung, thus confirming results from previous studies [4, 14]. The degree of PE was similar in the apical and basal lung lobes. Hypoxia-induced PE such as HAPE is thought to result from a strong increase in pulmonary capillary pressure due to a pronounced but uneven hypoxic pulmonary vasoconstriction [11, 34]. Patients with HAPE showed reduced levels in exhaled NO [28] and elevated levels of circulating ET-1 [9]. Vasodilators such as nifedipine or PDE-5 inhibitors have successfully been used for preventing or treating HAPE [31, 50, 55]. Reduction of ET-1 and improvement of NO levels have been described to be important mechanisms of RLX-mediated vasodilation [2, 25]. In hypoxic sheep, RLX administration significantly decreased pulmonary vascular resistance [60]. Therefore, we had expected RLX to attenuate or even to prevent PE formation. Contrasting to this expectation, RLX did not attenuate hypoxia-induced pulmonary injury. Neither PEI nor TNFα expression in hypoxic animals were reduced with RLX infusion. In particular, induction of PE in the NRLX-L group was unexpected. We assume that this was caused by unopposed pulmonary vasodilation. In the HRLX-L group, hypoxic pulmonary vasoconstriction may have counteracted the vasodilation and thus, restricted PE formation. In turn, in the HRLX-H group RLX-mediated vasodilation was predominant, thus rendering hypoxic pulmonary vasoconstriction less effective. This finding is in correspondence with a previous study in rats showing that β -adrenergic stimulation could induce pulmonary edema due to strong β-adrenergic pulmonary vasodilation [53]. Similarly, β-adrenergic agents administered for tocolysis can induce acute PE in pregnant women. This is a rare but severe complication that has repeatedly been described [61, 67, 73]. It indicates that exaggerated vasodilation, in particular in association with other edema-promoting factors, can result in formation of acute PE. The relation between vasodilation and PE formation corresponds well with the recognized pathophysiological concept of HAPE that uneven HPV and regional overperfusion in the lungs promote increased fluid filtration into pulmonary interstitium [11, 63, 71].

Interestingly, in RLX-infused animals, we observed a significant difference in PEI between the lung lobes with PE being more severe in the AL. Even in normoxia, RLX induced a significant PE in the AL but not in the BL. Of note, in the BL, PEI was in a similar range as in normoxic and hypoxic controls. Lung histology showed that RLX induced congestion in the lungs indicating pulmonary vasodilation, and this was associated with elevated *W*/*D* ratio. Both congestion and increased *W*/*D* ratio were only observed in RLX-treated animals but not in normoxic or hypoxic controls. We assume that lung perfusion was increased due to the vasodilatory effect of RLX. In the normal position of rats in their cage, the caudal part of their body is usually slightly elevated compared to the cranial part. Consequently, the apical parts of the lung are subject to gravity and thus, to higher hydrostatic pressure than the basal regions inducing strong overperfusion of the AL. This may account for the more severe PE in the AL of RLX-infused rats. Contrasting to our expectation, RLX did not diminish regional inhomogeneity in pulmonary perfusion but rather aggravated it. This may explain that RLX failed to prevent development of hypoxic PE. In addition, these hydrostatic effects of RLX may be amplified by a mismatch in the pump function of the two ventricles in hypoxia: While LV inotropic depression was not relieved with RLX, RVSP and inotropy were improved or even restored with RLX. This imbalance between the inotropic functions of RV and LV may be a further explanation why RLX was ineffective in preventing or attenuating hypoxia-induced PE.

Protein concentration in BALF was significantly elevated in hypoxic control rats, indicating a possible damage of the alveolo-capillary barrier. In the development of HAPE, strong increase of pulmonary capillary pressure can induce capillary wall stress leading to ultrastructural damage of the capillary wall and fluid and protein leakage into the alveoli [70]. Low-dose RLX infusion further increased BALF protein concentration, but this was not significant compared to hypoxic controls. We think that overperfusion of the AL may have aggravated capillary wall stress in this region. Of note, serum protein concentration was significantly elevated in hypoxic RLX-treated animals. The vasodilator RLX increases renal plasma flow (RPF) and consequently, GFR [19]. Even though the increase in RPF exceeds that of GFR, fluid filtration is enhanced with RLX [23]. We assume that enhanced filtration leads to hypovolemia and to a secondary increase in serum protein concentration. We suggest that high-dose RLX infusion might even have enhanced hypovolemia. This might have relieved pulmonary circulation, thus preventing disruption of capillary walls and protein leakage into alveoli.

The focus of these experiments was to study RLX effects on the development of hypoxic pulmonary edema and inflammation in the early stage of exposure to hypoxia. These processes were not prevented by RLX. As discussed above, RLX-induced vasodilation may rather promote PE formation. More detailed investigations on the molecular RLX effects such as on NO and ET-1 concentrations and on vascular contractility are necessary to explain the exaggerated RLX-induced vasodilation. Of particular interest would be future studies on RLX effects on the ET type B (ET_B_) receptor, the role of which in PE formation has been controversely discussed [13, 16].

RLX has been shown to exert anti-inflammatory and antifibrotic effects on several tissues including the lungs, but these effects occurred after RLX administration over several days [45, 66]. We think that prolonged treatment with RLX can reduce inflammation, and thus promote regression of edema but this has to be investigated in future studies.

### Limitations

The study was originally designed to comprise four animal groups: two control groups (NCtrl, HCtrl) and two RLX groups (NRLX-L, HRLX-L). As RLX has a bell-shaped dose–response curve [19], we primarily chose a low RLX dose for the experiments. First results showed that administration of RLX at this low dose to hypoxic rats induced slight improvement of hypoxic PE. Therefore, we added a group of hypoxia-exposed rats receiving high-dose RLX infusion. As these results were negative (no improvement of hypoxic PE compared to HCtrls and, moreover, no improvement of LV inotropic parameters), we refrained from adding a normoxic RLX-H group.

For the first time, we detected marked differences in PEI between apical and basal lung lobes. This had never been observed in hypoxic animals receiving NaCl infusion. Lung *W*/*D* ratio is largely insensitive to regional differences in PE distribution. A limitation is that congestion index as an indicator of pulmonary vasodilation was only assessed over the total lung without differentiation between apical and basal lobes. We would suppose that distribution of congestion might be similar to PE distribution.

The experimental design with external infusion pumps and catheters required single housing of the animals. Studies on rats have shown that single housing may induce stress and can promote the production of proinflammatory cytokines such as TNF α [40]. As all animals groups in this study were housed under equal conditions, the differences in TNF α expression can be considered to be real effects of hypoxia and RLX.

The present experiments were performed on female rats only to ensure comparability with previous studies [4, 14, 53, 54]. As this was the first study on RLX effects on formation of hypoxic PE, we focused on female animals. Although male animals also express RLX, their responses to RLX infusion might differ from those of female animals. A comparison between female and male rats should be reserved to further studies.

## Conclusions

The main finding of this study was that the vasodilator RLX cannot prevent or attenuate development of hypoxia-induced pulmonary injury characterized by moderate interstitial edema and inflammation. The results indicated that RLX induced vasodilation, thus counteracting hypoxic pulmonary vasoconstriction, which is a main pathogenic factor in PE formation. However, pulmonary vasodilation resulted in overperfusion of the lungs. This was aggravated by increased hydrostatic pressure due to gravity effects as indicated by significantly stronger PE in the apical lobe compared to the basal lobe. In addition, differential RLX effects on RV and LV might have resulted in an inotropic imbalance between RV and LV, which may have further increased pulmonary congestion and edema.
